# Supplementary material for: Individualized Prognostic Prediction of the Long-Term Functional Trajectory in Pediatric Acquired Brain Injury
Source: J Pers Med. 2021 Jul 18;11(7):675. doi: 10.3390/jpm11070675 (PMC8305391; doi:10.3390/jpm11070675)
Supplement: Supplementary file 1 [file jpm-11-00675-s001.zip › jpm-1271924-supplementary.pdf]

SUPPLEMENTARY MATERIAL

Individualized prognostic prediction of the long-term functional trajectory in pediatric acquired brain injury.

Erika Molteni[1], Marta Bianca Maria Ranzini[1], Elena Beretta[2*], Marc Modat[1] and Sandra Strazzer[2].

1. School of Biomedical Engineering & Imaging Sciences, King's College London, London, United Kingdom.
2. Acquired Brain Injury Unit, Scientific Institute IRCCS E. Medea, Bosisio Parini, Italy

Supplementary material 1. Flowchart of the patients' enrollment and inclusion in the study.

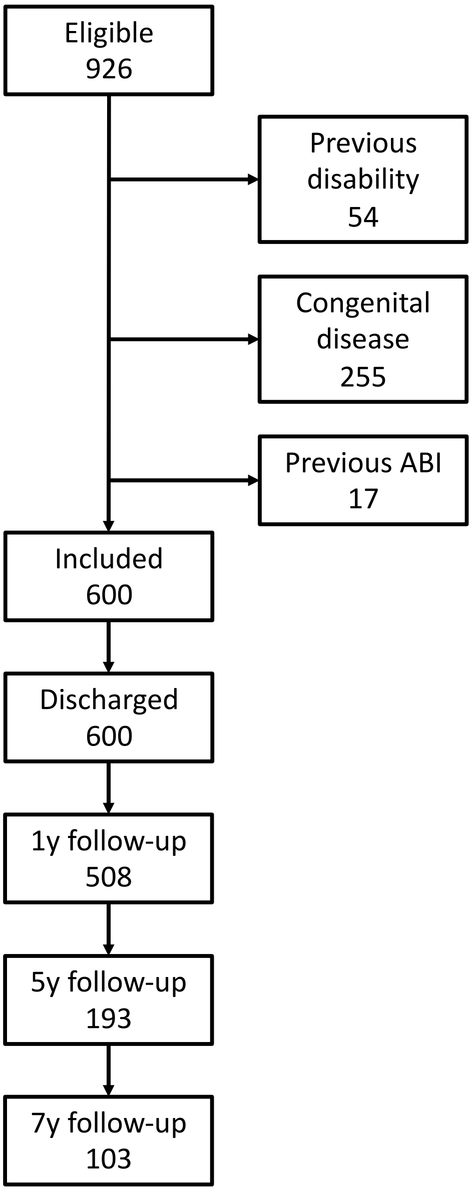

Supplementary material 2. Age distribution of the cohort.

Histogram of the age distribution. The vertical axis reports the number of patients up to the specified age, in months.

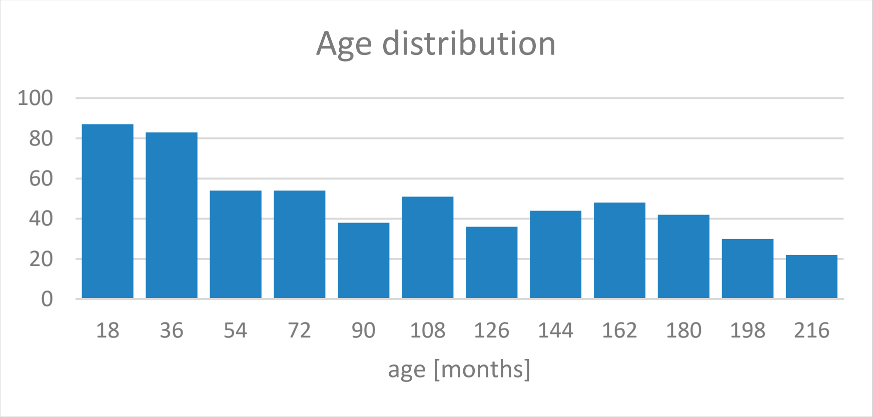

Supplementary material 3. Comparison of cohort characteristics over the first, middle and last 5-year periods.

Age at trauma, age at admission, and length of stay are reported as mean (standard deviation), and tested through ANOVA over the three time periods. Days of coma and GCS are reported as median and [interquartile range], and tested through Kruskal-Wallis non-parametric test. Chi square test is applied to etiology. Significant tests are indicated in bold in the last column (p-val<0.05).
Over time, the patients admitted to the service were less in number and less severe according to GCS. They also stayed longer, on average, in the rehabilitation center.

	**before 2009**	**2009 - 2013**	**after 2013**	**p-val**
N	258	219	122	
age at trauma [months]	93 (63)	86 (62)	88 (59)	0.4542
age at admission [months]	94 (63)	86 (55)	92 (60)	0.5901
days of coma	16 [7-40]	15 [4-45]	15 [3-40]	0.4850
GCS	5 [4-7]	6 [3.5-8]	6 [4-8]	**0.0316**
aetiology [TBI; non-TBI]	141; 117	81; 138	52; 70	**0.0004**
LOS [days]	113 (73)	129 (78)	123 (59)	**0.0199**
FIM at first admission	18 [18-35]	21 [18-43]	18 [18-29]	0.1662
FIM at first discharge	33 [18-79]	40 [18-88]	49 [18-87]	0.4582

Supplementary Table S1. *Cohort characteristics over the first, middle and last 5-year periods.*

Supplementary material 4. Rehabilitation program and Functional Independence Measure.

Rehabilitation program. All the patients were proposed a post-acute rehabilitation treatment according to the protocol used in our Intensive Rehabilitation Unit[1], based on their clinical and functional conditions and at least five days a week (supplementary figure 1). The rehabilitation program consisted of two stages: 1) an in-stay intensive treatment during the sub-acute phase of ABI, with multidisciplinary face-to-face therapies, daily sessions (Monday to Friday), and duration of

treatment spanning from a minimum of 3 weeks to a maximum of 7 months (supplementary table 2), and 2) a standardized home-based treatment over the chronic phase of ABI[2] (supplementary table 3). Rehabilitation was delivered according to a team-based multidisciplinary standardized program, developed in compliance with the International Classification of Function of the World Health Organization.

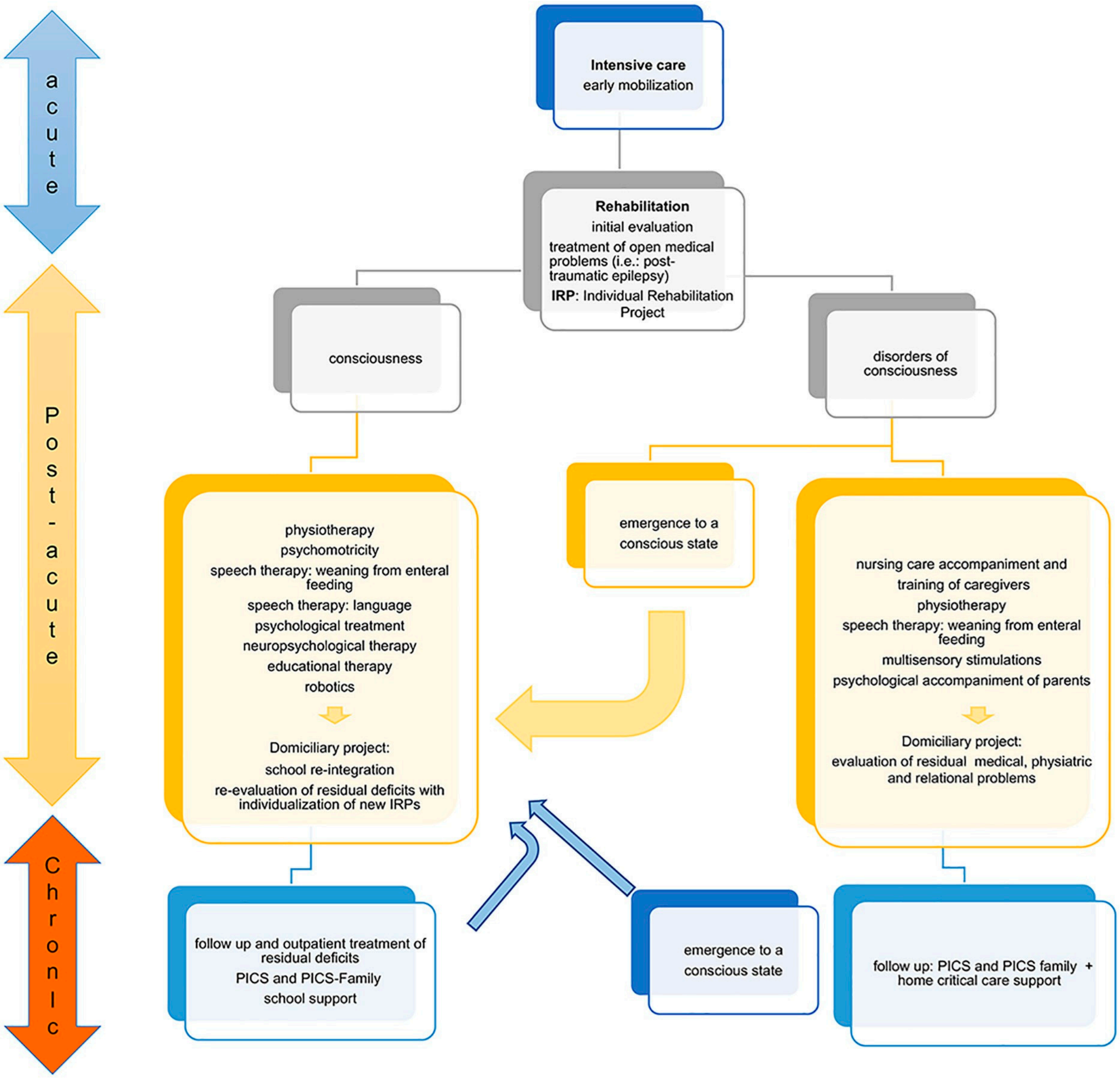

Supplementary Figure S1. *Flowchart of clinical recovery and main outcomes during the rehabilitation process. The patient who comes out of intensive care is assessed to set up the Individual Rehabilitation Project (IRP). The state of consciousness conditions two different rehabilitation paths. In the conscious patient, the goal is to recover the impaired functions. In the patient with a disorder of consciousness the main goal is to stimulate the state of consciousness. The proposed approach, arising from the experience of E. Medea Institute (Bosisio parini, Italy), could be generalizable to other services. Image reproduced with authors' permission from Nacoti et al.*[1]

<u>In-stay intensive treatment</u>. Each patient with ABI was appropriately assessed to set up the Individual Rehabilitation Project (IRP). Depending on the state of consciousness, they entered two different rehabilitation paths. Patients with a disorder of consciousness were treated through an intensive rehabilitation program towards the main goal to stimulate the state of consciousness; conscious patients received therapies towards the goal to recover the impaired functions. The IRPs were re-

evaluated and updated weekly because patients can emerge from a disorder of consciousness at any stage of the disease (although more frequently during the post-acute than chronic phase). Progress of children demonstrating functional recovery was re-evaluated during the development and modification of the functional deficit, and new rehabilitation objectives, that required new intensive paths, were set. Standard weekly schedule is summarized in supplementary table 2.

**Patients with disorders of consciousness**		
physical therapy	two daily sessions lasting 45 minutes each.	Physical rehabilitation aims at preventing secondary damage such as muscle retractions and joint deformities, enhancing normalization of muscular tone, and correcting posture.
oro-facial therapy	one daily session lasting 45 minutes.	Oro-facial therapy aims at restoring normal feeding.
cognitive-behavioral therapy	two to three daily sessions, lasting 10-20 minutes each, as described in detail in previous studies by our group.[3,4]	Since the early days of recovery, cognitive-behavioral stimulations may help patients to reinforce their adaptive responses – either spontaneous or elicited by multisensory stimulations - and rebuild their behavioral repertoire.
psychoeducational intervention	once a week	Psychoeducational intervention aimed at involving the patients' families in psycho-stimulation.
**Conscious patients**		
physical therapy	two daily sessions lasting 45 minutes each.	Physical rehabilitation aims improve movement to resume walking and manipulation.
speech therapy	one daily session lasting 45 minutes.	Speech therapy aims to improve oral communication or to initiate augmentative alternative communication.
neuropsychological therapy	one daily session lasting 45 minutes.	This therapy aims to stimulate different cognitive domains (attention, memory, problem solving and executive functions).
psycho-educational therapy	one daily session lasting 45 minutes.	This therapy, done in small groups, aims to reduce behavioral problems and to improve social relationships.
caregivers' psychoeducational intervention	twice a month	It aimed at involving the patients' families and raise awareness of the rehabilitation process and residual problems.

Supplementary Table S2. *Structure of the standard in-stay treatment program.*

Standardized home-based treatment. Outpatient rehabilitation services included physical (PT), occupational (OT), and speech therapy (ST)[5–7]. Follow-up assessments were preformed yearly by rehabilitation physicians. Families/caregivers were involved in the interventions, and they were trained to act in first person to intervene for improving attention, memory, executive functioning, and emotional/behavioral functioning in daily life and home setting. Differences in treatment provision were however necessary for different age-ranges (supplementary table 3).

Discharge from hospital	- Goal setting<br>- Contact of the local healthcare services<br>- Survey of the available resources<br>- (Remote) meeting with the local services.<br>- Upon full availability of the resources, set up of the

	community-based or home-based treatments locally, and transfer of care.
Long term care	- Weekly local treatments based on standardized elements of care (exercises and activities).
Monitoring	- Remote teleconsultation to local resources by the professional in charge of the patient at our center (monthly, every three months, or upon request dependent on the phase disease).<br>- Yearly follow-ups at the center.

Supplementary Table S3. *Structure of the standard home-based program.*

Functional Independence Measure (FIM). The Functional Independence Measure (FIM) was designed for an intended population of patients with functional mobility impairments. The tool is used to assess a patient's level of disability as well as a change in patient's status in response to rehabilitation or medical intervention. The FIM uses the level of assistance an individual needs to grade functional status from total independence to total assistance. The instrument is composed of 3 subscales rating Mobility, Self-Care and Cognitive abilities, containing 18 items covering 6 domains of functioning: activities of daily living, sphincter control, transfers, locomotion, communication, and social cognition. The need for assistance in activities of daily living is rated on a seven-point ordinal scale (1=complete dependence; 7=complete independence) for single items. Scores falling below 6 require another person for supervision or assistance. The minimum score overall is 18, corresponding to a score of 1 at all items and patients' complete dependence. The maximum is 126, indicating complete independence. Inter-Rater Reliability of FIM has been established at an acceptable psychometric performance (Intraclass co-relation coefficients ranging from 0.86 to 0.88). The concurrent validity with Barthel Index (ICC > 0.83) has shown strong construct validity between items of the two scales.

Analogously, the WeeFIM is a paediatric adaptation of the adult FIM. It equivalently summarises the need for assistance in activities of daily living on a seven-point ordinal scale over the 18 items and six domains. Scores are then age-normalised with respect to published normative data, acquired from normally developing children.

Supplementary material 5. Data preprocessing and descriptive mixed modeling.

Preprocessing. We conducted preliminary correlation analysis between demographic and clinical variables, to identify independent information. We selected as covariates: gender, age at event, etiology, age at admission, length of stay (LOS), days of coma, need of decompressive craniotomy/neurosurgery (DC/N), and insurgence of epilepsy during in-stay. Data at first admission and discharge were available from all patients. Missing data at follow-ups are depicted in table 2 in the main text. Covariate data are complete.

Mixed models. We converted raw FIM scores into WeeFIM, according to age-matched normative data. All FIM/WeeFIM data were then normalized between 0 and 1, and demeaned. To linearize the outcome variable WeeFIM over the time-points, we tested logarithmic, exponential and hyperbolic tangent transformations on the corresponding time values. We optimized the time transformation parameters by minimizing the sum of standard errors from single-subject fits. We selected an initial *unconditional model* relating time and WeeFIM/FIM total or domain scores as outcome variables. Time, measured continuously from first admission up to the 7th year follow-up, entered the model at level 1 (within persons). The origin for recovery curve analysis was set to first admission. The scale was based on the number of years between the origin and the assessment. Linear, quadratic, and cubic effects of time were included as fixed effects (and later interpreted as *growth parameters*); only linear time contributions were considered as random effects, as in Pretz et al.[8] and Hart et al.[9] This baseline model was subsequently employed to test the contribution of each covariate.

Covariate data entered the model at level 2 (between persons). Four variables were included as categorical fixed effects: gender, etiology, DC/N and insurgence of epilepsy. Three variables entered as continuous fixed effects: age at event, LOS and days of coma. The relative significance of adding each covariate and its interaction with each time component to the unconditional model was assessed using F-tests (supplementary figure 2).

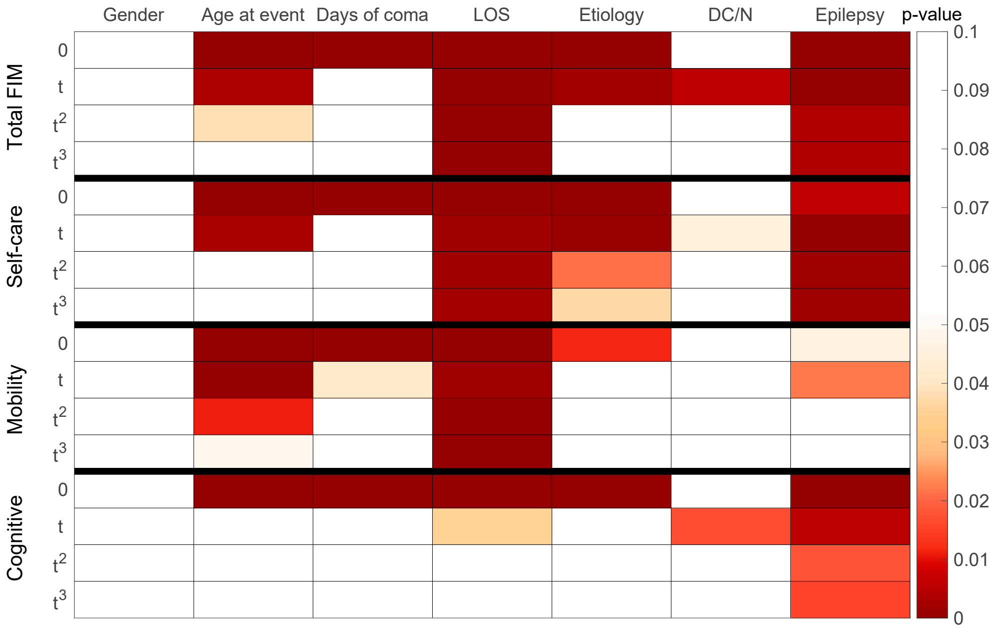

Supplementary Figure S2. *Results of the single covariate selection. The table represents the p-value for F-tests obtained in the single covariate analysis. Only the significant intercept (0) and time interaction (t, t^2, t^3) terms were selected for the final parsimonious multivariable model (here represented by the colored boxes, based on the associated p-value). Significance threshold is set at $p = 0.05$. Gender was not significant and was excluded from further analyses. Age at event, days of coma, LOS, etiology and presence of epilepsy were significantly related to all FIM domains. Overall, LOS and epilepsy were found to moderate the trajectory parameters up to the highest growth parameter. DC/N was significantly related to the total FIM scores and to selfcare and cognitive domains, but not mobility, with the first rate parameter.*

To determine a parsimonious multivariable model, a *conditional model* was designed, which combined all significant covariate-growth parameter associations (supplementary figure 3). Python (v.3.7) and *statsmodels* (v.10.1) library were used.

Beta coefficients in final minimal model

		Gender	Age at event	Days of coma	LOS	Infectious et.	Anoxic et.	Ischemic et.	Tumour exc. et.	Other et.	DC/N	Epilepsy
Total FIM	0		2.61e-01	-3.74e-01	-3.12e-01	NS	NS	4.02e-01	NS	NS		NS
	t		NS		2.27e-01	NS	-1.14e-01	NS	NS	NS	-6.05e-02	-3.04e-01
	t^2		NS		-8.72e-02							NS
	t^3				8.75e-03							NS
Self-care	0		2.70e-01	-3.18e-01	-3.01e-01	NS	NS	NS	NS	NS		NS
	t		NS		NS	NS	NS	NS	NS	NS	-5.73e-02	-3.88e-01
	t^2				NS	NS	NS	NS	NS	NS		NS
	t^3				NS	NS	NS	NS	NS	NS		NS
Mobility	0		1.63e-01	-1.53e-01	-2.72e-01	NS	NS	NS	NS	NS		NS
	t		NS	-6.53e-02	NS							-6.00e-02
	t^2		NS		NS							
	t^3		NS		NS							
Cognitive	0		2.36e-01	-4.28e-01	-2.64e-01	NS	NS	3.08e-01	NS	NS		NS
	t				NS						NS	NS
	t^2											NS
	t^3											NS

Supplementary Figure S3. *Results of the final parsimonious model. The table reports the model coefficients (β) obtained from the fit of the final parsimonious model for total FIM and its three domains. Values are color-coded according to the strength and the direction of the effect for the intercept (0) and time-interaction (t, t^2, t^3) terms per covariate. Red indicates positive relation between the model term and the outcome; blue indicates a negative correlation. Empty cells represent terms that did not enter the final model, while NS indicates terms that entered the final model but resulted non-significant when combined with the other terms (significance threshold at p=0.05, Bonferroni correction for multiple testing is applied). Etiology covariates comprise: infectious, anoxic, ischemic, tumor and other mixed causes.*

Results. The minimal models for the total FIM and its domains included 600 participants who had complete data on all covariates. The 4 growth parameters were interpreted as follows: (1) an intercept that predicts the FIM score at first admission (start of intensive rehabilitation), (2) a first rate parameter that estimates the tangent to the curve at first admission (initial slope of the curve, at the intercept), (3) a second rate parameter that estimates the curvature of the FIM trajectory, and (4) a third rate parameter which estimates the depth of the curvature or other changes in shape.

The final models, applied to the total FIM trajectories and to FIM domains time-course, put in evidence that older age related to initial higher scores at total FIM and at all domains at admission, but with no steeper tangent (initial slope). Longer period of coma associated with initial lower scores at total FIM and at all domains at admission; it also related to less steep tangent (i.e. slower initial improvement) at mobility FIM. Analogously, longer LOS related to initial lower scores, and to reduced curvature at total FIM; however, longer LOS also related to steeper tangent at initial recovery. Anoxic etiology presented with less steep tangent to the total FIM curve, indicating progress slower than TBI course. Ischemic etiology showed higher initial total and cognitive scores, indicating more favorable (cognitive) functioning at baseline than TBI. DC/N and presence of epilepsy were

associated with slower tangent at admission, for total FIM and the selfcare domain. Epilepsy also related to slower tangent in the mobility domain (supplementary figure 3).

Interpretation. Mixed models were applied to provide preliminary description of the factors influencing the recovery trajectory over long term. Peculiar to pediatrics, older children were admitted with initial higher scores at FIM, but their progress was not faster than the one observed in younger peers. Previous studies showed that children younger than 6 years have worse long-term outcome after an anoxia[10] and stroke[11], and faster gross motor recovery was found in older children.[12] In our study, WeeFIM normalization might have produced under-compensation for young ages, thus biasing the assessments of the youngest patients towards lower scores. However, our result also matches independent observations,[12] and our previous findings[13] that children under 3 years of age struggle more to recover effectively in short times, with overall worse outcome.

Days of coma and LOS can both be regarded as proxy of injury severity. Thus, it is not surprising that longer coma and LOS were associated with lower FIM scores at first admission. However, similarly to what observed in adults,[9] longer coma associated with slower improvements at mobility FIM during in-stay, probably due to delayed initiation of effective physiotherapy, reduced recovery potential, and flooring effects of the FIM tool itself.[2] In contrast, longer LOS related to faster improvement, supporting the effectiveness of the intensive rehabilitation effort. Of note, no therapy was withdrawn due to poor improvement in this cohort, but parents opted out in some cases as described.

Regarding etiology, our data confirm that initial functional scores are more favorable in ischemic stroke than in TBI, especially in the cognitive domain; while anoxia associates with overall slower early recovery, in line with a recent review.[14]

Among the complicating factors, DC/N and epilepsy related to slower early progress in total and selfcare FIM. Also, early recovery in epileptic patients proceeded slower than average in the mobility domain. This agrees with studies on children with ischemic stroke,[15] showing that the presence of epileptic seizures predicts worse cognitive outcome. Similarly to adult studies,[16] we could not find any effect for gender.

Supplementary material 6. Survival analysis.

Definitions.

Survival was defined as the return to the center for follow-up assessment.

Death was defined as any patient deceased (either in the hospital or at home) after the first admission to the rehabilitation center. Deaths in the intensive care units or in the acute phase of injury are not computed here, as patients qualified for the study at admission to the post-acute rehabilitation center.

Exit due to recovery was defined as the indication, given by the medical professionals to the family, to interrupt the yearly attendance of follow-up visits at the center. This indication was provided to patients who had excellent and stable clinical and functional recovery.

Non-recommended opt-out was defined as the families' unilateral decision to interrupt the yearly attendance of follow-up visits at the center, in the absence of an indication to do so by the

professionals. Main causes of *non-recommended opt-out* were the long distances between the center and the usual address of the patient, availability of nearer services, and families' professional constraints. Perception of unsatisfactory provision of care at the center was not a main cause.

Survival analysis. Survival analysis was conducted to investigate the characteristics of patients who remained engaged with the rehabilitation service and continued to attend the follow-ups, as well as to assess the risk to drop out of the service. *Survival* was defined as the return to the center for follow-up assessment. Kaplan-Meier survival estimate and Nelson-Aalen cumulative hazard estimate were calculated with *death*, *exit due to recovery* and *non-recommended opt-out* as causes of failure (supplementary figure 4). Risk was set to start at first discharge. Stratified log-rank tests were conducted on gender, etiology, epilepsy and DC/N for testing the equality of the survivor functions by group. Accelerated failure time model was applied with gender, age at event, days of coma, LOS, etiology, DC/N and epilepsy as covariates (Gompertz and Weibull regressions were tested, obtaining negligible differences). Cox proportional hazards model was applied with the same covariates for confirmation. 'Linearity' and 'proportional hazard' assumptions were verified by calculating Martingale and log-scaled Schoenfeld residuals respectively. STATA v.16.1 software was used.

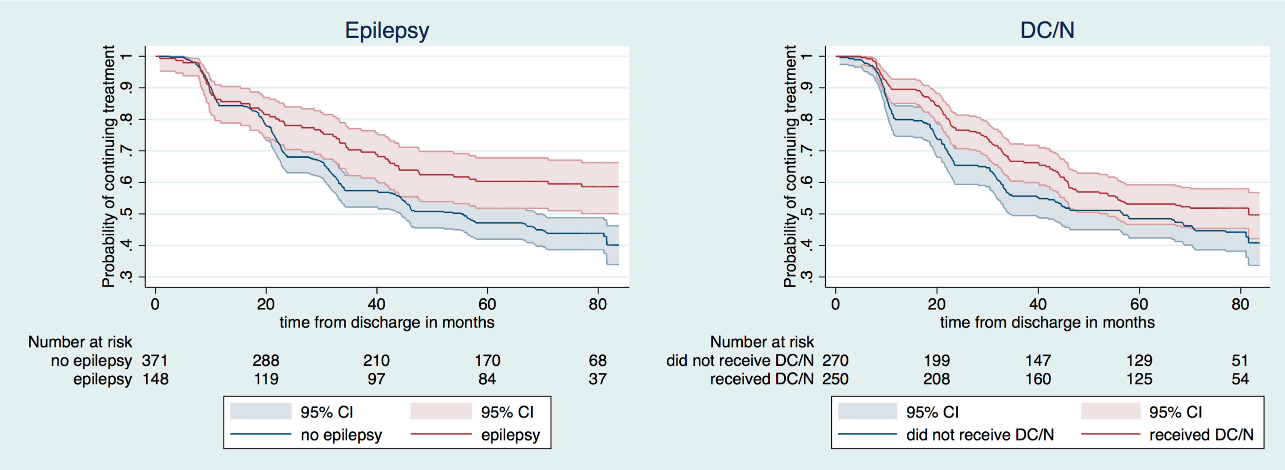

Supplementary Figure S4. *Kaplan-Meier survival estimates for patients with and without epilepsy, and for patients who received and did not receive DC/N. Patients who manifested epilepsy or received DC/N showed higher probability to remain inside the rehabilitation service.*

Results. Survival analysis showed that the rate of cumulative failure gradually reduced over time, indicating that patients who remained in the rehabilitation service for longer were gradually less likely to drop out. Patients with epilepsy (Chi-sq=1692, p<<0.0001) and those who received DC/N (Chi-sq=1688, p<<0.0001) were more likely to remain into the service for longer (supplementary figure 4). Patients with older age and shorter LOS had increased risk to drop out of the rehabilitation service (c>1, p<0.001 in all cases).

Interpretation. We examined the characteristics of the patients lost to follow-ups, to ascertain the presence of any longitudinal bias in the dataset. Bias can cause over- or underestimation of the effects of the rehabilitation treatments over time. We observed that younger patients, those with longer LOS, and those with complexities (i.e., DC/N and epilepsy) tended to engage with the rehabilitation service

for longer time. Thus, recovery might be underestimated in this study. This contrasts with adult studies showing that the unhealthiest participants are the most likely to be lost to follow-up and excluded from the longitudinal models, thus causing potential overestimation of improvements.[17–20] Differences in age and criteria for cohort selection,[21] and the unlike supportive role of family and school in the pediatric context possibly account for this dramatic difference in long-term participation.

Supplementary material 7. Clustering and prediction (extended).

Clustering. A generalized structural equation model (SEM) was designed to aggregate similar recovery trajectories (supplementary figure 5), and to identify responders to treatment and non-responders.

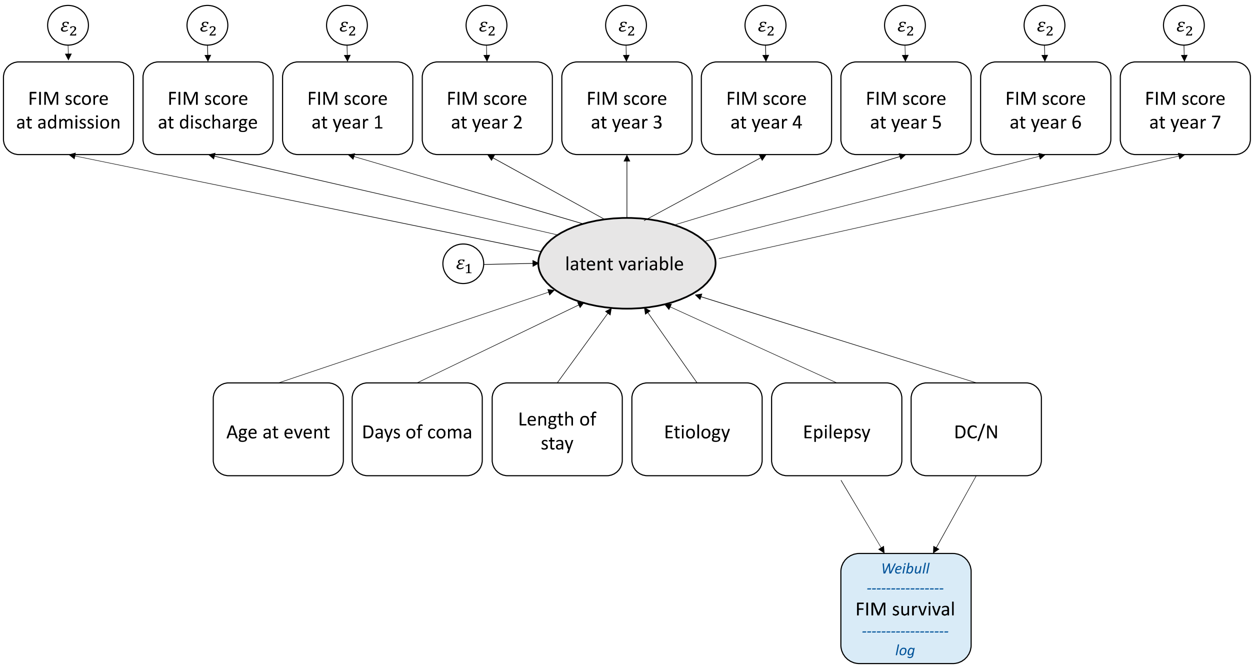

Supplementary Figure S5. *Generalized structural equation model employed to cluster the trajectories. The model includes the explanation of the outcome trajectory, one latent class for the prediction of the trajectory type from the covariates, and the characterization of the survival into the rehabilitation service over time, based on epilepsy and DC/N information.* ε_i *indicates the model residual errors.*

Prediction. Single-subject prediction of the long-term trajectory membership was performed through a logistic regression model, using demographic, clinical and functional data available by the day of first discharge. The days from event to discharge were used in place of the days of coma, if shorter. Only FIM data at first admission and discharge were included for prediction. Prediction was run in cross-validation on unseen cases only. The entire method is summarized graphically in supplementary figure 6.

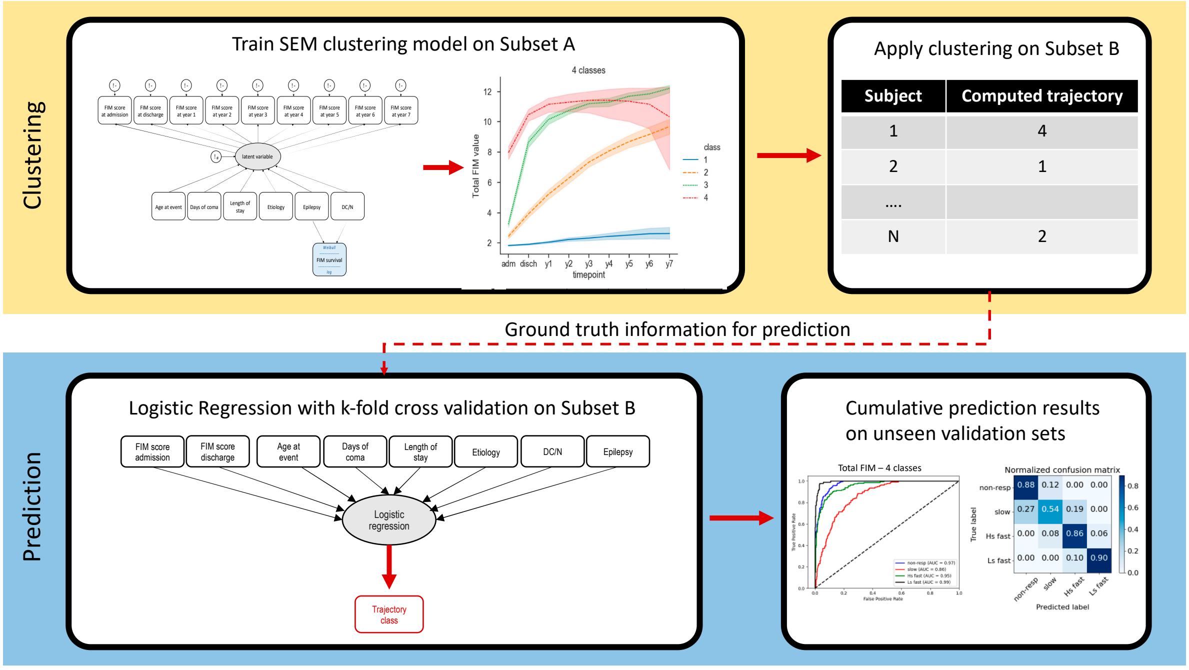

Supplementary Figure S6. *Workflow of the prediction method on 4-class clusters. We trained the clustering method on data subset A, and then we applied it to subset B. Then, individualized cluster assignments were used as ground truth for validation of the class prediction through logistic regression on subset B. The procedure was repeated for 2- and 3-class clusters, and with inversion of the data subsets, thus training the clustering model on subset B, estimating ground truth clustering on subset A, and then performing prediction on the same subset A.*

Supplementary material 8. Table of FIM values over time.

Scores are reported as median (IQR) from the whole cohort.

	Total FIM	Selfcare FIM	Mobility FIM	Cognition FIM
Admission	18 (18-38)	8 (8-12)	5 (5-6)	5 (5-17)
Discharge	38 (18-85)	11 (8-33)	8 (5-27)	15 (5-27)
Year 1	48 (19-100)	16 (8-42)	14 (5-31)	17 (6-29)
Year 2	60 (21-105)	23 (8-42)	19 (5-32)	18 (7-30)
Year 3	67 (20-104)	25 (8-43)	20 (5-32)	20 (7-30)
Year 4	69 (20-105)	27 (8-45)	24 (5-32)	20 (7-30)
Year 5	79 (19-112)	30 (8-48)	26 (5-33)	22 (6-30)
Year 6	69 (18-106)	25 (8-45)	22 (5-33)	19 (5-30)
Year 7	51 (18-105)	18 (8-45)	17 (5-34)	16 (5-28)

Supplementary material 9. Non-parametric effect sizes. Cohen's d based on medians.

	Admission	Discharge	Year 1	Year 2	Year 3	Year 4	Year 5	Year 6	Year 7
**Two classes**									
Responders vs. non-responders	0.83	1.86	2.05	2.13	2.20	2.20	2.21	2.21	2.23
**Three classes**									
Fast responders vs. non-responders	1.30	1.95	2.04	2.07	2.20	2.27	2.26	2.39	2.56
Fast responders vs. slow responders	1.20	1.73	1.74	1.43	1.68	1.64	1.48	1.33	1.10
Slow responders vs. non-responders	0.33	1.32	1.52	1.90	1.84	2.00	2.03	2.08	2.10
**Four classes**									
High-start fast responders vs. non-responders	1.90	2.15	2.31	2.49	2.75	2.84	2.86	2.92	3.37
Low-start fast responders vs. non-responders	1.00	1.78	2.00	2.07	2.16	2.18	2.22	2.46	2.56
Slow responders vs. non-responders	0.00	1.36	1.69	1.90	1.92	2.07	2.06	2.09	2.19
High-start fast responders vs. slow responders	1.84	1.92	1.94	1.94	2.10	1.96	1.90	1.52	1.05
Low-start fast responders vs. slow responders	1.00	1.58	1.77	1.75	1.80	1.64	1.80	1.56	1.53
High-start fast responders vs. low-start fast responders	1.70	0.88	0.75	0.72	0.63	0.60	0.25	0.21	0.17

Interpretation: d<0.01 is very small; 0.01<d<0.20 is small; 0.20<d<0.50 is medium; 0.50<d<0.80 is large; 0.80<d<1.40 is very large; d>1.40 is huge

References of the supplementary material.

1. Nacoti M, Fazzi F, Biroli F, Zangari R, Barbui T, Kochanek PM. Addressing Key Clinical Care and Clinical Research Needs in Severe Pediatric Traumatic Brain Injury: Perspectives From a Focused International Conference. *Frontiers in Pediatrics*. 2021;8. doi:10.3389/fped.2020.594425
2. Beretta E, Molteni E, Galbiati S, Stefanoni G, Strazzer S. Five-year motor functional outcome in children with acquired brain injury. Yet to the end of the story? *Developmental Neurorehabilitation*. Published online 2018. doi:10.1080/17518423.2017.1360408
3. Liscio M, Adduci A, Galbiati S, et al. Cognitive-behavioural stimulation protocol for severely brain-damaged patients in the post-acute stage in developmental age. *Disability and Rehabilitation*. Published online 2008. doi:10.1080/09638280701257023

4. Villa F, Colombo K, Pastore V, et al. LOCFAS-assessed evolution of cognitive and behavioral functioning in a sample of pediatric patients with severe acquired brain injury in the postacute phase. *Journal of Child Neurology*. 2015;9:1125-1134. doi:10.1177/0883073814553798
5. Laatsch L, Dodd J, Brown T, et al. Evidence-based systematic review of cognitive rehabilitation, emotional, and family treatment studies for children with acquired brain injury literature: From 2006 to 2017. *Neuropsychological Rehabilitation*. 2020;30(1). doi:10.1080/09602011.2019.1678490
6. Chevignard MP, Toure H, Brugel DG, Poirier J, Laurent-Vannier A. A comprehensive model of care for rehabilitation of children with acquired brain injuries. *Child: Care, Health and Development*. 2010;36(1). doi:10.1111/j.1365-2214.2009.00949.x
7. Jimenez N, Symons RG, Wang J, et al. Outpatient rehabilitation for medicaid- insured children hospitalized with traumatic brain injury. *Pediatrics*. 2016;137(6). doi:10.1542/peds.2015-3500
8. Pretz CR, Kozlowski AJ, Dams-O'Connor K, et al. Descriptive modeling of longitudinal outcome measures in traumatic brain injury: A national institute on disability and rehabilitation research traumatic brain injury model systems study. *Archives of Physical Medicine and Rehabilitation*. Published online 2013.
9. Hart T, Kozlowski AJ, Whyte J, et al. Functional recovery after severe traumatic brain injury: An individual growth curve approach. *Archives of Physical Medicine and Rehabilitation*. Published online 2014. doi:10.1016/j.apmr.2014.07.001
10. Shaklai S, Peretz Fish R, Simantov M, Groswasser Z. Prognostic factors in childhood-acquired brain injury. *Brain Injury*. 2018;32(5):533-539. doi:10.1080/02699052.2018.1431843
11. de Montferrand C, Vassel-Hitier J, Yvon-Chaou E, Câmara-Costa H, Dellatolas G, Chevignard M. Language and cognitive outcomes after childhood stroke: Theoretical implications for hemispheric specialization. *Cortex*. Published online 2019. doi:10.1016/j.cortex.2019.07.020
12. Kelly G, Mobbs S, Pritkin JN, et al. Gross Motor Function Measure-66 trajectories in children recovering after severe acquired brain injury. *Developmental Medicine and Child Neurology*. Published online 2015. doi:10.1111/dmcn.12592
13. Recla M, Galbiati S, Pastore V, et al. Children sustaining a severe acquired brain lesion before age 3 years: a follow-up study at 1 year from insult. *Brain Injury*. Published online 2019. doi:10.1080/02699052.2018.1539763
14. Mackay MT, Steinlin M. Recent developments and new frontiers in childhood arterial ischemic stroke. *International Journal of Stroke*. Published online 2019. doi:10.1177/1747493018790064
15. Anderson V, Darling S, Mackay M, et al. Cognitive resilience following paediatric stroke: Biological and environmental predictors: Resilience following paediatric stroke. *European Journal of Paediatric Neurology*. Published online 2020. doi:10.1016/j.ejpn.2019.11.011
16. Lu J, Roe C, Sigurdardottir S, Andelic N, Forslund M. Trajectory of Functional Independent Measurements during First Five Years after Moderate and Severe Traumatic Brain Injury. *Journal of Neurotrauma*. Published online 2018. doi:10.1089/neu.2017.5299
17. Malec JF, Ketchum JM, Hammond FM, et al. Longitudinal Effects of Medical Comorbidities on Functional Outcome and Life Satisfaction after Traumatic Brain Injury: An Individual Growth Curve Analysis of NIDILRR Traumatic Brain Injury Model System Data. *Journal of Head Trauma Rehabilitation*. Published online 2019. doi:10.1097/HTR.0000000000000459
18. Thompson HJ, McCormick WC, Kagan SH. Traumatic brain injury in older adults: Epidemiology, outcomes, and future implications. *Journal of the American Geriatrics Society*. Published online 2006. doi:10.1111/j.1532-5415.2006.00894.x

19. Kumar RG, Olsen J, Juengst SB, et al. Comorbid Conditions among Adults 50 Years and Older with Traumatic Brain Injury: Examining Associations with Demographics, Healthcare Utilization, Institutionalization, and 1-Year Outcomes. *Journal of Head Trauma Rehabilitation*. Published online 2019. doi:10.1097/HTR.0000000000000470
20. Hammond FM, Corrigan JD, Ketchum JM, et al. Prevalence of Medical and Psychiatric Comorbidities Following Traumatic Brain Injury. *Journal of Head Trauma Rehabilitation*. Published online 2019. doi:10.1097/HTR.0000000000000465
21. Hernán MA, Hernández-Díaz S, Robins JM. A structural approach to selection bias. *Epidemiology*. Published online 2004. doi:10.1097/01.ede.0000135174.63482.43
